# Konsensusempfehlungen zur regionalen fächerübergreifenden Standardisierung der MRT-Diagnostik bei Multipler Sklerose im Großraum Essen

**DOI:** 10.1007/s00115-023-01531-2

**Published:** 2023-08-18

**Authors:** Mark Stettner, Mike P. Wattjes, Karlgeorg Krüger, Refik Pul, Michael Fleischer, Ute Achnitz, Heike Agne, Kathlen Bach, Ralf Berkenfeld, Ulrike Bongartz, Anja Brüggemann, Lars Burgsmüller, Joseph Cohnen, Cornelius Deuschl, Anke Friedrich, Patrizia Graziano, Jana Hackert, Beate Hapig, Andrea Henkel, Heike Henrich, Mechthild Hükelheim-Görden, Luder Kratsch, Danuta Kytzia, Rotem Lanzman, Philipp Heusch, Claus Laufenburg, Susanne Merguet, Uwe Metz, Michael Montag, Michel Obeid, Ahmet Ornek, Sören Peters, Theo Plajer, Jürgen Plassmann, Heiko Pump, Birgit Rauchfuss-Hartych, Marcus Paul Reinboldt, Katja Seng, Michael Stauder, Anna-Katharina Wettig, Anna Wolters, Sedat Yilmam, Christoph Kleinschnitz

**Affiliations:** 1 Klinik für Neurologie, Universitätsmedizin Essen, Universität Duisburg-Essen, Hufelandstr. 55, 45147 Essen, Deutschland; 2 Center for Translational Neuro- and Behavioral Sciences (C-TNBS), Universitätsklinikum Essen, Hufelandstraße 55, 45147 Essen, Deutschland; 3 Institut für Diagnostische und Interventionelle Neuroradiologie, Medizinische Hochschule Hannover, Hannover, Deutschland; 4 Diavero Diagnosezentrum, Heidbergweg 22–24, Essen, Deutschland; 5 Praxis für Neurologie, Psychiatrie und Psychotherapie, Bochum, Deutschland; 6 Nervenstark Praxis für Neurologie und Psychiatrie, Essen, Deutschland; 7 Neurologische Praxis, Neukirchen-Vluyn, Deutschland; 8 Praxis für Neurologie, Köln, Deutschland; 9 Radiologie am Kennedyplatz, Essen, Deutschland; 10 Evangelische Kliniken Essen-Mitte gGmbH, Henricistraße 92, Essen, Deutschland; 11 Radiologie der Ruhrradiologie Essen, Rüttenscheider Straße 191, Essen, Deutschland; 12 Institut für Diagnostische und Interventionelle Radiologie und Neuroradiologie, Universitätsmedizin Essen, Essen, Deutschland; 13 Zentrum für ambulante Neurologie, Essen, Deutschland; 14 Radiologie am Stern, Essen, Deutschland; 15 Radiologie und Nuklearmedizin Am Kennedyplatz, Essen, Deutschland; 16 Dr. med. Theo Plajer & Dr. med. Heike Henrich, Fachärzte für Radiologie in Essen-Borbeck, Essen, Deutschland; 17 Praxis für Psychotherapie, Psychiatrie und Neurologie, Drosselstraße 20, Essen, Deutschland; 18 Neuropraxis am EKO, Virchowstraße 39, Oberhausen, Deutschland; 19 Praxis Dr. Kytzia, Altenessener Straße 208, Essen, Deutschland; 20 Radiologie MH, Schulstraße 13, Mülheim an der Ruhr, Deutschland; 21 MVZ Radios, Luise-Rainer-Str. 6–10, Düsseldorf, Deutschland; 22 Praxis Dr. Merguet, Gerichtsstraße 32, Essen-Borbeck-Mitte, Deutschland; 23 Ruhrradiologie Essen Henricistraße, Henricistraße 40, Essen, Deutschland; 24 Klinik für Radiologie, Universitätsklinikum Münster, Albert-Schweitzer-Campus 1, Münster, Deutschland; 25 Praxis Dr. Obeid – Praktischer Arzt, Kriemhildstraße 8, Gelsenkirchen, Deutschland; 26 Ruhrradiologie Gelsenkirchen, Zum Ehrenmal 21, Gelsenkirchen, Deutschland; 27 Radiologische Gemeinschaftspraxis Mülheim, Schulstraße 13, Mülheim an der Ruhr, Deutschland; 28 Radiologie Bredeneyer Tor, Am Alfredusbad 8, Essen, Deutschland; 29 Neuroradiologie, Alfried Krupp Krankenhaus, Rüttenscheid, Alfried-Krupp-Straße 21, Essen, Deutschland

**Keywords:** Vereinheitlichung, Sequenzen, Bildgebung, Verlaufskontrolle, Leitlinie, Harmonization, Sequences, Imaging, Follow-up, Guideline

## Abstract

Der Magnetresonanztomographie (MRT) kommt bei der Diagnostik und Verlaufsbeobachtung der Multiplen Sklerose (MS) eine herausragende Bedeutung zu. Jedoch ist zwischen niedergelassen Neurologen, (neuro)radiologischen Praxen, Krankenhäusern oder spezialisierten MS-Zentren nur selten eine enge interdisziplinäre Zusammenarbeit etabliert. Es fehlen insbesondere standardisierte MRT-Protokolle zur Bildakquisition sowie etablierte Qualitätsparameter, die die Vergleichbarkeit von MRT-Aufnahmen garantieren. Das ist jedoch eine grundlegende Voraussetzung für den effektiven Einsatz der MRT in der Versorgung von MS-Patienten, z. B. im Rahmen der Diagnosestellung oder des Therapiemonitorings. Zur Adressierung dieser Herausforderungen erarbeitete im Rahmen eines Pilotprojektes im Großraum Essen eine Gruppe aus Neurologen und (Neuro)radiologen unter Anwendung eines modifizierten mehrstufigen Delphi-Prozesses und auf Basis der aktuellsten wissenschaftlichen Untersuchungen einen Konsensvorschlag zur Standardisierung der Bildakquisition, Interpretation und Befundübermittlung und zur Verbesserung der interdisziplinären Zusammenarbeit. Die Empfehlung berücksichtigt medizinische, wirtschaftliche, zeitliche und praktische Aspekte der MRT-Bildgebung bei der MS. Das Modell der interdisziplinären Zusammenarbeit zwischen Radiologen und Neurologen mit dem Ziel der regionalen Standardisierung der Magnetresonanztomographie könnte als Vorbild für andere Regionen Deutschlands dienen, um die MRT-Bildgebung bei der MS zu optimieren.

## Abstract (en)

Magnetic resonance imaging (MRI) is of exceptional importance in the diagnostics and monitoring of multiple sclerosis (MS); however, a close interdisciplinary cooperation between neurologists in private practice, (neuro)radiological practices, hospitals or specialized MS centers is only rarely established. In particular, there is a lack of standardized MRI protocols for image acquisition as well as established quality parameters, which guarantee the comparability of MRI records; however, this is a fundamental prerequisite for an effective application of MRI in the treatment of MS patients, e.g., for making the diagnosis or treatment monitoring. To address these challenges a group of neurologists and (neuro)radiologists developed a consensus proposal for standardization of image acquisition, interpretation and transmission of results and for improvement in interdisciplinary cooperation. This pilot project in the metropolitan area of Essen used a modified Delphi process and was based on the most up to date scientific knowledge. The recommendation takes the medical, economic, temporal and practical aspects of MRI in MS into consideration. The model of interdisciplinary cooperation between radiologists and neurologists with the aim of a regional standardization of MRI could serve as an example for other regions of Germany in order to optimize MRI for MS.

## Einleitung

Die Magnetresonanztomographie (MRT) des Gehirns und des Rückenmarkes hat in den vergangenen Jahrzehnten sowohl bei der Diagnosestellung als auch bei der Verlaufskontrolle der Multiplen Sklerose (MS) kontinuierlich an Relevanz gewonnen [1–3]. Darüber hinaus liefern die MRT-Befunde zu Beginn der Erkrankung wichtige prognostische Informationen hinsichtlich Langzeitbehinderung, kognitiver Beeinträchtigung und individueller Krankheitsprogression. Im Rahmen der Therapieüberwachung kommt der MRT, neben der Detektion entzündlicher Krankheitsaktivität, auch bei der Früherkennung von Komorbiditäten und Nebenwirkungen moderner Immuntherapien wie beispielsweise opportunistische Infektionen eine grundlegende Rolle zu.

Obwohl standardisierte MRT-Protokolle bei der MS schon länger international konsentiert werden [4], sind enge Kooperationen zwischen Radiologen und Neurologen im klinisch Alltag selten und die Bildgebung ist kaum vereinheitlicht. Dies hat potenziell negative Auswirkungen auf die Qualität der Versorgung von Menschen mit MS. So schränkt beispielhaft die fehlende Vergleichbarkeit von MRT-Bildern im Verlauf die Aussagekraft bezüglich der paraklinischen Krankheitsaktivität ein und fehlende präzise klinische Angaben und Fragestellungen sowie Voraufnahmen erschweren die Befunderstellung durch den Radiologen. Neben den potenziell negativen Folgen für die Krankenversorgung sprechen darüber hinaus medizinökonomische Aspekte für eine (regionale) Harmonisierung der MRT-Bildgebung bei MS. Nicht nur die MRT-Bildgebung als Kostenfaktor selbst, sondern auch MRT-basierte therapeutische Entscheidungen hinsichtlich hochpreisiger Immuntherapien machen die optimale Nutzung der „Ressource MRT“ durch eine enge Zusammenarbeit zwischen Radiologen und Neurologen unabdingbar.

Die Autorengruppe berichtet hier über ein Projekt zur Standardisierung und Harmonisierung der MRT-Bildgebung bei MS-Patienten auf regionaler Ebene. Dazu wurden im Großraum Essen gemeinsam zwischen Neurologen und (Neuro)radiologen im Rahmen mehrerer strukturierter Treffen Konsensuskriterien verabschiedet. Im Falle einer dauerhaften erfolgreichen Umsetzung könnte dieses Pilotprojekt Vorbild für andere Regionen in Deutschland sein und dazu beitragen, die Versorgung von MS-Patienten zu verbessern.

## Projektbeschreibung und Methoden

Ein Fragenkatalog zur Standardisierung der Bildakquisition, Interpretation, Befundübermittlung und zu weiteren Aspekten zur Verbesserung der interdisziplinären Zusammenarbeit wurde im Expertenkreis diskutiert. Zur Konsensfindung wurde die modifizierte Delphi-Methode angewendet, eine strukturierte Kommunikationstechnik, entwickelt als Vorhersagemethode unter Fachexperten [12], welche in der wissenschaftlichen Konsensfindung häufig Anwendung findet [13]. Grundlage des Konsenses waren die aktuellen wissenschaftlichen Untersuchungen zur Magnetresonanztomographie bei der MS [4] sowie praktische Überlegungen zur technischen Verfügbarkeit und der Durchführbarkeit in Anbetracht wirtschaftlicher und zeitlicher Aspekte. Ein Konsensvorschlag wurde als angenommen angesehen, wenn zwei Drittel der Experten diesem zustimmten.

Zur Zusammenstellung des Expertenkreises wurden 2018 alle Neurologen und Radiologen aus dem Großraum Essen angeschrieben (97 Empfänger) und Ziel wie auch das geplante Vorgehen erläutert. 43 Experten beteiligten sich am Verfahren, hierunter waren 19 Neurologen und 20 Radiologen aus 20 verschiedenen Niederlassungen und 5 verschiedenen Kliniken unterschiedlicher Versorgungsstufen (z. B. Unikliniken und städtische Kliniken). Zwischen 2018 und 2023 fanden sich die Experten 6‑mal in Präsenz oder virtuell zusammen und erarbeiteten einen Konsens.

## Konsens zu Diagnosestellung und Therapieüberwachung bei der MS

Es bestand in der Konsensusgruppe Einigkeit darüber, dass folgende Aspekte zu Diagnosestellung und Therapieüberwachung bei der MS zur Konsensfindung vorausgesetzt werden können: Zur Diagnosestellung der MS werden die 2017 revidierten McDonald-Kriterien herangezogen [8]. Die MRT-Bildgebung ermöglicht die Dokumentation der örtlichen und zeitlichen Dissemination mit einer einzigen MRT-Untersuchung bei simultaner Anwesenheit schrankengestörter und nichtschrankengestörter Läsionen in MS-typischen Lokalisationen [8]. Auch wenn eine zeitliche Dissemination auf Basis der MRT-Befunde nicht besteht, kann der Liquorbefund (Nachweis liquorspezifischer oligoklonaler Banden) bei MR-tomographisch nachweisbarer örtlicher Dissemination zur Diagnosestellung beitragen [8]. Es bestand Konsens darüber, dass MRT-Verlaufsuntersuchungen notwendig sind, um auch subklinische Krankheitsaktivität zu erfassen und die Effektivität der eingeleiteten Therapie festzustellen. Diese Verlaufsuntersuchungen sollten in aller Regel in 12-monatigen Intervallen durchgeführt werden. Bei Therapieumstellung sollte nach ca. 6 Monaten ein sog. Rebaselining-MRT (MRT des Gehirns ohne Kontrastmittelgabe) durchgeführt werden. Unabhängig von den routinemäßigen Verlaufskontrollen können nach klinischer Maßgabe bei Schubereignissen oder verdachtsgetrieben MRT-Untersuchungen erfolgen.

Es bestand Konsens darüber, dass neuere Parameter der MRT durch Messung der Hirnatrophie die neurodegenerative Komponente der MS erfassen. So ist in der Literatur bekannt, dass MS-Patienten im Vergleich zu gesunden Menschen eine deutlich gesteigerte jährliche Volumenminderung der grauen und weißen Substanz aufweisen [9–11]. Volumetrische Parameter der MRT haben einen prädiktiven Wert hinsichtlich der Langzeitbehinderung und der individuellen Krankheitsprogression. Konsens bestand in der Gruppe darüber, dass aktuell die Volumetrie aufgrund der Notwendigkeit einer hohen Standardisierung der Bildakquisition, verschiedener beeinflussender Faktoren (z. B. Lifestyle-Faktoren, antiinflammatorische Therapie) und der fehlenden Validierung der Daten für die Therapieentscheidung noch nicht in der klinischen Routine als etabliert betrachtet werden kann.

## Konsens zu allgemeinen Aspekten der MRT-Untersuchung von MS-Patienten

Die MRT-Untersuchung sollte bei der Verdachtsdiagnose einer MS zur Steigerung von Spezifität und Sensitivität einheitlichen Standards entsprechen. In der klinischen Routine ist häufig nicht eindeutig definiert, wann ein MS-MRT-Protokoll vom Radiologen durchgeführt werden soll. Es bestand Konsens darüber, dass dieses Protokoll zumindest dann ausgeführt wird, wenn der Zuweiser auf dem Überweisungsschein MS bzw. ein entsprechendes Synonym vermerkt. Synonyme bzw. Abkürzungen für die MS sind in der Tab. 1 aufgeführt.

**Table 1:** 

Abkürzung	Bedeutung
MS	Multiple Sklerose
ED	Encephalomyelitis disseminata
RRMS	„Relapsing remitting MS“ (schubförmige MS)
SPMS	Sekundär chronisch-progrediente MS
PPMS	Primär progrediente MS
–	Entzündliche ZNS-Erkrankung

## Konsens zur MRT des Gehirns

Die magnetische Feldstärke ist eine der wichtigsten MRT-Akquisitionsparameter mit Einfluss auf die Läsionsdetektion im Gehirn. Im Vergleich zu 1,5 Tesla (T) können Untersuchungen bei 3 T mehr MS-Läsionen detektieren. Jedoch gibt es bisher keinen Hinweis darauf, dass eine Untersuchung bei 3 T im Vergleich zu 1,5 T tatsächlich zu einer verbesserten bzw. früheren MS-Diagnose führt. Auch vor dem Hintergrund der begrenzten Verfügbarkeit von 3 T-Geräten bestand daher Konsens darüber, dass eine Feldstärke von 1,5 T zur Diagnosestellung und Verlaufskontrolle der MS ausreichend ist. Es sollten jedoch für die Verlaufsuntersuchungen die Feldstärke der Voraufnahme angewendet werden, um eine Vergleichbarkeit der Bilder zu gewährleisten.

**Figure 1:**
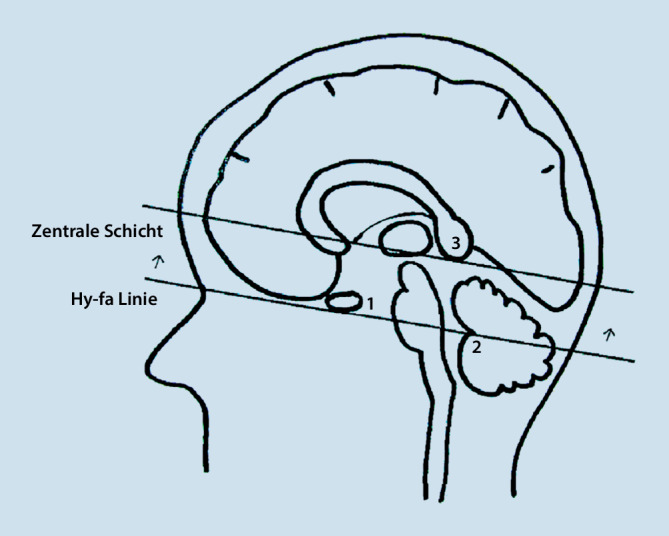

Auch sollte zur besseren Vergleichbarkeit der Bilder eine standardisierte Repositionierung (fixe Angulierung bzw. Kippung) der Schnittführung festgelegt werden. Aufgrund der weiten Verbreitung bestand Konsens zur bevorzugten Angulierung entlang der HYFA-Linie, welche den Unterrand der Hypophyse mit dem Fastigium des IV. Ventrikels verbindet (Abb. 1). Alternativ kann die sog. „subcallosal line“ als Angulierungslinie verwendet werden, welche das Genu anterius und das Splenium des Corpus callosum verbindet. Eine isolierte Bildgebung der Sehnerven ist im Allgemeinen nicht erforderlich und spezifischen Fragestellungen vorbehalten.

Zur Planung sollte eine exakt ausgerichtete koronare Sequenz auf dem axialen Lokalizer und als Leitstruktur die Verbindungslinie der inneren Gehörgänge angewendet werden. Das hiermit akquirierte koronare Bild sollte der Planung einer exakten sagittalen Schnittführung mit dem Interhemisphärenspalt als Leitstruktur dienen. Auf dem sagittalen Lokalizer ist die Positionierung der axialen Messsequenzen zu planen, zur Orientierung dient hier die Verbindungslinie der unteren Begrenzung von Genu und Splenium des Corpus callosum. Die zentralste Schicht der axialen Schichten ist parallel zu dieser Verbindungslinie zu positionieren. Die midsagittale Schicht ist die Referenz für alle weiteren Verlaufsuntersuchungen.

Grundsätzlich sollte ein minimales Zeitintervall von 5 min zwischen der Applikation von Kontrastmittel und dem Akquirieren der kontrastmittelgestützten Sequenzen eingehalten werden. Es sollten nur noch makrozyklische gadoliniumbasierte Kontrastmittel verwendet werden, da in ihnen – im Vergleich zu linearen Kontrastmitteln – das Gadolinium besser gebunden vorliegt, was eine Anreicherung im Körper weniger wahrscheinlich macht ([14]; Tab. 2).

**Table 2:** 

1,5 T-MRT ausreichend (Diagnosestellung und Verlauf)
Angulierung entlang der HYFA-Linie (alternativ „subcallosal line“)
Midsagittale Schicht Referenz für Verlaufsuntersuchungen
Mindestens 5 min zwischen Applikation des KM und Akquisition der Post-KM-Sequenzen
Nur makrozyklische Gd-basierte Kontrastmittel verwenden

Es bestand Konsens darüber, dass die in Tab. 3 angeführten Sequenzen bei der Erstuntersuchung durchgeführt werden sollten. Alle 2‑D-Aufnahmen sind mit einer Schichtdicke von 3 mm und einer In-plane-Auflösung von 1 × 1 mm zu akquirieren. Für 3‑D-Sequenzen wird eine gemessene Voxelgröße von 1 × 1 × 1 mm empfohlen. Bei der Erstuntersuchung sollte stets die KM-Gabe vor der FLAIR-Sequenz und vor der axialen T2-Sequenz erfolgen; so kann die Zeit der T2-Sequenz genutzt werden, um eine ausreichende Verzögerung vor Anfertigung der Post-KM-T1-Sequenz zu gewährleisten. Für T1w-Sequenzen können statt TSE/SE-Sequenzen auch Gradienten-Echo-Sequenzen angefertigt werden. Sowohl 2‑D- wie auch 3‑D-Sequenzen sind möglich, 3‑D-Sequenzen sind jedoch zu bevorzugen.

**Table 3:** 

T1-gewichtete Aufnahmen („fast spin echo“) axial
Kontrastmittelgabe
FLAIR (vorzugsweise 3‑D, Voxelgröße 1 × 1 × 1 mm, alternativ axial und sagittal)
T2-gewichtete Aufnahmen axial
Post-KM-T1-gewichtete Aufnahmen („fast spin echo“), alternativ 3‑D-T1-gewichtete Aufnahmen mit axialer Rekonstruktion

## Konsens zur MRT des Rückenmarks

Bei der Erstuntersuchung zur Abklärung einer MS sollte stets eine MRT der kompletten spinalen Achse durchgeführt werden. Die Untersuchung sollte das gesamte Myelon einschließlich des Conus medullaris abbilden und wenn möglich als Gesamtaufnahme durchgeführt werden (keine Abschnittsuntersuchungen). Eine Untersuchung mit 1,5 T und 3 mm Schichtdicke ist hierfür ausreichend. Die Gabe von Kontrastmittel ist bei der Erstuntersuchung zur Klärung der Diagnose MS sinnvoll. Zeigen sich Läsionen im Myelon, so sollten weitere axiale Aufnahmen durch die Läsion und Post-KM-T1-gewichtete Aufnahmen generiert werden. Spinale MRT Untersuchungen zur Beurteilung der Therapieeffektivität werden im Allgemeinen nicht empfohlen, können aber erwogen werden, wenn dies individuell für sinnvoll erachtet wird. Klinische Konstellationen, bei welchen eine spinales MRT auch zur Verlaufsbeobachtung indiziert sein kann, werden in den MAGNIMS-CMSC-NAIMS-Empfehlungen aufgeführt [4]. Für etwaige Verlaufsuntersuchungen, ist die Gabe von Kontrastmittel in den meisten Fällen nicht zielführend und sollte vermieden werden (Tab. 4).

**Table 4:** 

T2-gewichtete Aufnahmen: sagittal
Protonengewichtete Sequenzen (Doppelecho) oder STIR: sagittal
Ggf. T2-Aufnahmen axial durch die Läsionen
T1-gewichtete Aufnahmen: sagittal nach Kontrastmittelgabe
Post-KM axiale T1-Aufnahmen mit KM (nur bei gesicherten Läsionen)

## Konsens zu MRT-Verlaufsuntersuchungen

Die MRT-Verlaufsuntersuchungen nach gesicherter Diagnose einer MS sollten analog zur Erstuntersuchung erfolgen, jedoch in der Regel ohne erneute Gabe von Kontrastmittel. Die Indikation der Kontrastmittelgabe sollte vom behandelnden Neurologen vor dem Hintergrund der sich daraus ergebenen klinischen Konsequenz dokumentiert werden und interdisziplinär mit dem verantwortlichen (Neuro)radiologen besprochen werden, da letzterer die Endverantwortung für die Kontrastmittelgabe trägt. Neben der magnetresonanztomographischen regelmäßigen Verlaufskontrolle zur Feststellung der Krankheitsaktivität dient die MRT dem Sicherheitsmonitoring hinsichtlich therapieassoziierter Komplikationen, wie beispielsweise zur frühen Feststellung einer progressiven multifokalen Leukenzephalopathie (PML) unter einer Therapie mit Natalizumab. Hierzu sollte in der Anforderung der Vermerk „verkürztes PML-Protokoll“ angegeben sein. Das Protokoll macht nur Sinn, wenn die Untersuchung alle 3 bis 6 Monate durchgeführt wird. Die Sequenzen für dieses verkürzte Protokoll finden sich in Tab. 5.

**Table 5:** 

T2-gewichtete Aufnahmen axial
FLAIR-3‑D (mit axialen Rekonstruktionen)
DWI-Aufnahmen

## Konsens zu den Mindestangaben des Neurologen bei Anforderung der Bildgebung

Um die Arbeit des Neuro‑/Radiologen bei der Befundung, insbesondere bei Verlaufsuntersuchungen, zu vereinfachen und eine hohe Befundqualität zu ermöglichen, sollte der Anfordernde obligatorische Mindestangaben zur Fragestellung, zu der zugrunde liegenden Symptomatik, der aktuellen Krankheitsaktivität sowie zur MS-Verlaufsform zur Verfügung stellen (Tab. 6).

**Table 6:** 

MS-Verlaufsform? Klinisch stabil? Wenn Schub – welche Symptome?
Jahr der Erstdiagnose und der Erstmanifestation?
Voraufnahmen vorliegend? Datum?
Aktuelle Therapie?
„Hochrisiko“-Vortherapien und ggf. weitere Gründe für Immunsuppression bzw. -defizienz?

## Konsens zu den Befundtexten

Für viele Neurologen besteht keine Möglichkeit, die zahlreichen magnetresonanztomographischen Untersuchungen selbst eingehend zu sichten und zu befunden. Häufig ist im ambulanten Setting selbst für Erstuntersuchungen nicht die Zeit, die Aufnahmen persönlich detailliert in Augenschein zu nehmen. Aufgrund dieser Umstände kommt den radiologischen Befundtexten entscheidende Bedeutung zu.

Allgemein sind für den Neurologen Angaben zu Größe, Konfiguration, Lokalisation und Anzahl der T2-Läsionen relevant, da die quantitative Bewertung der T2-Läsionslast einen guten prognostischen Parameter für die Krankheitsaktivität und den Verlauf der Erkrankung darstellt. Auch die Bewertung schrankengestörter Läsionen nach Gabe von Kontrastmittel, wie z. B. im Rahmen der Erstuntersuchung, ist zur Abschätzung der Krankheitsaktivität relevant. Neben anderen klinischen Aspekten leitet sich aus diesem Parameter die Therapiewahl ab.

Auch die visuelle semiquantitative Abschätzung der Hirnatrophie kann unter bestimmten Umständen therapeutische Entscheidungen beeinflussen. Die magnetresonanztomographischen Verlaufsuntersuchungen bewerten insbesondere die Zunahme der Läsionslast und das Vorhandensein schrankengestörter Läsionen. Die im Befundtext aufzugreifenden Bewertungsaspekte sind in Tab. 7 zusammengestellt.

**Table 7:** 

Beschreibung der pathologischen Befunde, welche für die klinische Fragestellung relevant sind
Zahl der T2-Läsionen und der schrankengestörten Läsionen, Topographie, Größe, Konfiguration (mit Bezug auf die MS-Charakteristika)
Qualitative Bewertung der T2- und T1-Läsionslast
Semiquantitative visuelle Einschätzung der Hirnatrophie
Für und Wider bez. der Diagnose MS oder einer alternativen Diagnose
Weitere inzidentelle oder ungewöhnliche Befunde und Einschätzung hinsichtlich klinischer Relevanz
Verlaufsuntersuchungen: – Zahl der KM-affinen Läsionen – Zahl der neuen oder vergrößerten T2-Läsionen

Auch in Zukunft wird den Befundtexten weiterhin ein hoher Stellenwert zukommen, wenngleich mittelfristig einheitliche Softwarelösungen verfügbar sein werden, die zuverlässig Bilder miteinander vergleichen, Läsionen quantifizieren, vermessen und anatomisch zuordnen. Der Einsatz derartiger Software hat derzeit aber noch nicht in den Routinebetrieb Einzug gehalten.

## Zusammenfassung und Ausblick

Man kann davon ausgehen, dass in den nächsten Jahren weitere Bildgebungsparameter zur Diagnostik und Verlaufskontrolle bei der MS in die klinische Praxis eingeführt werden. Dazu gehören die Erfassung volumetrischer Daten (Messung der Hirn- und Rückenmarksatrophie), ggf. unterstützt durch Techniken der künstlichen Intelligenz, sowie die Quantifizierung mikrostruktureller Veränderungen einschließlich neuronaler Reparaturmechanismen (z. B. Remyelinisierung) unter spezifischen Therapien [5–7]. Dadurch wird die Rolle der MRT bei der MS noch wichtiger werden. Diese neuen Techniken werden jedoch höchstwahrscheinlich nicht sofort in der Breite verfügbar sein, sondern sich zunächst auf größere Zentren beschränken. Allerdings ist es essenziell, dass auch bei limitierten Ressourcen (z. B. im niedergelassenen Bereich) eine evidenzbasierte Versorgung von MS-Patienten stattfinden kann. Dazu können solche Pilotprojekte auf regionaler Ebene beitragen. Wir haben einen strukturierten interdisziplinären Konsens zwischen Neurologen und (Neuro)radiologen im Großraum Essen erarbeitet, um die MRT-Bildgebung bei MS zu vereinheitlichen, ohne dabei wirtschaftliche Aspekte und Effizienzbetrachtungen außer Acht zu lassen. Nach erfolgreicher Implementierung werden wir nun in einem kontinuierlichen Prozess und weiteren Treffen die Einhaltung dieser Konsensuskriterien nachhalten. Zudem sollen weitere Konstellationen diskutiert werden, bei welchen eine Vereinheitlichung des MR-diagnostischen Vorgehens nützlich wäre, wie z. B. dem radiologisch isolierten Syndrom.
